# Correlation between disability and MRI findings in lumbar spinal stenosis: A prospective study of 109 patients operated on by decompression

**DOI:** 10.3109/17453674.2011.566150

**Published:** 2011-04-05

**Authors:** Freyr G Sigmundsson, Xiao P Kang, Bo Jönsson, Björn Strömqvist

**Affiliations:** ^1^Department of Orthopedics, Clincal Sciences Lund, Lund University, Skåne University Hospital, Lund; ^2^Department of Orthopedic Surgery, Blekinge Hospital, Karlshamn, Sweden

## Abstract

**Background and purpose:**

MRI is the modality of choice when diagnosing spinal stenosis but it also shows that stenosis is prevalent in asymptomatic subjects over 60. The relationship between preoperative health-related quality of life, functional status, leg and back pain, and the objectively measured dural sac area in single and multilevel stenosis is unknown. We assessed this relationship in a prospective study.

**Patients and methods:**

The cohort included 109 consecutive patients with central spinal stenosis operated on with decompressive laminectomy or laminotomy. Preoperatively, all patients completed the questionnaires for EQ-5D, SF-36, Oswestry disability index (ODI), estimated walking distance and leg and back pain (VAS). The cross-sectional area of the dural sac was measured at relevant disc levels in mm^2^, and spondylolisthesis was measured in mm. For comparison, the area of the most narrow level, the number of levels with dural sac area < 70 mm^2^, and spondylolisthesis were studied.

**Results:**

Before surgery, patients with central spinal stenosis had low HRLQoL and functional status, and high pain levels. Patients with multilevel stenosis had better general health (p = 0.04) and less leg and back pain despite having smaller dural sac area than patients with single-level stenosis. There was a poor correlation between walking distance, ODI, the SF-36, EQ-5D, and leg and back pain levels on the one hand and dural sac area on the other. Women more often had multilevel spinal stenosis (p = 0.05) and spondylolisthesis (p < 0.001). Spondylolisthetic patients more often had small dural sac area (p = 0.04) and multilevel stenosis (p = 0.06).

**Interpretation:**

Our findings indicate that HRQoL, function, and pain measured preoperatively correlate with morphological changes on MRI to a limited extent.

MRI plays a central role in the diagnosis of spinal stenosis. Despite this, the correlation between MRI characteristics and clinical symptoms remains elusive as a considerable number of asymptomatic subjects have MRI-verified spinal stenosis (Boden et al. 1990). The relationship between the hard pathomorphological data as seen on MRI and the more subjective data from accepted outcome tools in terms of HRLQoL, functional status, and pain is unknown but is clinically relevant.

The absolute reduced cross-sectional area that gives neurological symptoms of central spinal stenosis has been estimated to be around 75 mm^2^ (critical size) (Schönström 1988) and some studies today use a value of 70–80 mm^2^ as a definition of spinal stenosis (Malmivaara et al. 2007). Since MRI is used for the preoperative planning, any correlation between MRI findings and preoperative symptoms and disability would be of interest

We therefore investigated the relationship between the minimal dural sac area (mm^2^), number of levels with stenosis, and spondylolisthesis in relation to preoperative subjective measures of disease in terms of: self reported walking distance, the visual analog scale (VAS) for leg and back pain, Oswestry disability index, the 4 physical domains of the SF-36, and the EQ-5D.

## Patients and methods

109 consecutive patients operated for central spinal stenosis with decompressive laminectomy or laminotomy with facet-sparing technique without concomitant fusion were included in the study. The operations were performed from 2000 through 2007 by 5 surgeons specialized in spinal surgery. The median patient age was 71 (34–89) years. 53 patients were male. All the patients were diagnosed and operated at the Department of Orthopaedic Surgery in Lund, Sweden. Preoperative MRI was performed on all patients and dural sac area and number of stenotic levels was evaluated. The “critical size” of 70 mm^2^ was used as the objective diagnostic criterion for spinal stenosis (Schönström 1988).

### MRI evaluation

All MRIs were evaluated by one of the authors (XK). The dural sac area (mm^2^) at the disc levels in the lumbar spine was measured on axial T1 images using a region of interest (ROI) application on a workstation specially designed for such purposes, using SECTRA software. Spondylolisthesis was measured in mm. Difficult measurements were discussed and a subset of 20 random cases were measured independently by 3 of the authors and the correlation between the observations was calculated.

### Preoperative symptoms

All patients had symptoms consistent with spinal stenosis: neurogenic claudication, persistent leg and/or back pain, and weakness and numbness in one or both legs.

The patients completed the Swedish Spine Register protocol (Strömqvist et al. 2009) including the Swedish version of the Oswestry disability index, the health-related quality of life EuroQol index (EQ-5D), the SF-36, the visual analog scale for low back and leg pain, and walking distance graded as follows: 1 (< 100 m), 2 (100–500 m), 3 (500–1,000 m), and 4 (> 1,000 m).

### SF-36

The medical outcome study short form was designed for group comparisons involving generic health concepts not specific for age, disease, or treatment group (Ware and Sherbourne 1992). The SF-36 measures both physical and mental health components over the preceding week, covering 8 dimensions (subscales): physical functioning (PF), social functioning (SF), role physical (RF), role emotional (RE), mental health (MH), vitality (VT), bodily pain (BP), and general health (GH). The item scores for each dimension were coded and summed and transformed into a scale from 0 (maximum disability) to 100 (no disability).

### Oswestry disability index (ODI)

The ODI is a 10-question low back-specific instrument designed to measure disability in spine patients (Fairbanks et al. 1980). The ODI (version 2.0) was introduced in the Swedish Spine Registry in 2003, so this questionnaire was completed only by the last 58 patients.

### EQ-5D

The EQ-5D is a standardized quality of life instrument to measure health outcomes. The instrument has 5 dimensions: mobility, self-care, daily activities, pain/discomfort, and anxiety/depression. Each dimension has 3 possible answers (no problem, some problem, or major problem) of which only 1 can be selected (EuroQol Group 1990).

### Visual analog scale (VAS)

Visual analog scale scores for leg and back pain were obtained on the preoperative day by measuring the distance in mm from the origin of a horizontal line (total 100 mm) and the point indicated by the patient as representing their level of pain during the previous week. Zero represented “no pain at all” and 100 represented “the worst pain imaginable”.

### Statistics

STATA 10 statistical software was used. Parametric tests were used when only SF-36 variables were involved in the analysis. When comparison with other variables was done, for which no assumption of normal distribution could be made, non-parametric tests were used (Mann-Whitney, Spearman rho). When investigating correlation between functional measures—HRLQoL and pain to minimal dural sac area—Pearson's correlation was performed, controlling for levels involved. Values of p < 0.05 were regarded as statistically significant. The interclass correlation coefficient (ICC) was used in the reability assessment of minimal dural sac area measurements.

### Ethics

The patient group was part of the Swedish Spine Register and as such had given consent for participation in this study. The Swedish Spine Register is the property of the Swedish Society for Spinal Surgery and is funded by the National Board of Health and Welfare (Strömqvist et al. 2009).

## Results

### MRI findings

A high to moderate correlation was found between observers (A, B, and C) measuring the subset of 20 patients (0.53 between A and B, 0.78 between A and C, and 0.78 between B and C), (Table 1). Interclass correlation coefficient was 0.67 (95% CI: 0.45–0.83; p < 0.001).

**Table 1.** MRI measurements by 3 observers (A, B, and C) of minimal dural sac area (mm^2^) in a randomly selected subset of 20 patients

A	B	C
40	47	38
17	58	32
42	30	40
18	29	26
35	23	29
31	38	30
17	40	33
66	55	48
38	38	30
55	36	35
30	48	32
43	53	70
56	46	61
15	36	25
54	50	47
44	34	32
62	45	58
22	43	37
82	68	76
65	78	77

The mean of the minimal dural sac area was 43 mm^2^ (SD 17, range 13–99), 40 mm^2^ (SD 16) in women and 46 mm^2^ (SD 18) in men (Table 2). 105 patients had minimal dural sac area below 70 mm^2^, most often at the L4–L5 level (63 patients), followed by the L3–L4 level in 36 patients. Minimal dural sac area was less often localized at the L2–L3 level (8 patients) followed by the L5–S1 level in 2 patients. 4 patients had a minimal dural sac area of > 70 mm^2^. 51 patients had 1 level with a dural sac area of < 70 mm^2^; the remaining 54 had 2 or more levels < 70 mm^2^. Mean number of levels with stenosis below 70 mm^2^ was 1.5 (SD 0.8) (Table 2).

**Table 2.** Patient demographics, EQ-5D, VAS leg and back, minimal dural sac area, and multilevel stenosis. Values are mean (SD)

	No. of patients	Age (years)	EQ-5D	SF-36 PF	SF-36 GH	SF-36 BP	SF-36 RP	ODI	VAS leg	VAS back	Min. area (mm^2^)	Multilevel (no. of pts)
Sex												
Women	56	73 (9)	0.35 (0.29)	26 (18)	60 (20)	27 (16)	11 (26)	46 (16)	69 (26)	53 (26)	40 (16)	30
Men	53	69 (11)	0.47 (0.28)	37 (22)	60 (20)	25 (14)	8 (15)	45 (14)	66 (21)	55 (26)	46 (18)	24
Total	109	71 (10)	0.41 (0.29)	31 (21)	60 (20)	26 (15)	10 (22)	46 (15)	68 (24)	54 (28)	43 (17)	56
Age group												
0–49	2	40 (8)	0.79 (0.13)	53 (11)	75 (11)	36 (7)	25	38	72	41 (28)	64 (2)	1
50–59	14	56 (2)	0.17 (0.18)	29 (13)	65 (21)	21 (10)	18 (36)	51 (11)	72 (19)	48 (29)	47 (17)	7
60–69	21	65 (3)	0.45 (0.29)	39 (20)	65 (16)	26 (10)	0	44 (15)	64 (23)	48 (29)	44 (17)	8
70–79	53	75 (3)	0.44 (0.30)	30 (21)	56 (22)	28 (19)	9 (18)	45 (18)	67 (27)	60 (27)	42 (17)	29
80–89	19	83 (3)	0.39 (0.29)	23 (25)	61(15)	24 (13)	16 (32)	46 (10)	69 (21)	48 (28)	38 (16)	9
Preop walking distance, m												
<100	51	72 (9)	0.31 (0.29)	27 (23)	62 (20)	26 (18)	10 (22)	50 (18)	67 (26)	55 (28)	44 (19)	23
100–499	39	71 (9)	0.42 (0.27)	34 (17)	60 (18)	25 (13)	10 (25)	43 (13)	68 (23)	50 (29)	41 (16)	23
500–999	12	65 (15)	0.66 (0.19)	40 (17)	60 (23)	31 (11)	10 (18)	39 (12)	69 (20)	59 (27)	59 (27)	6
>1000	3	73 (14)	0.70 (0.02)	43 (21)	63 (15)	31 (10)	0	39 (8)	72 (17)	61 (11)	45 (20)	0

EQ-5D: health-related quality of life EuroQol index; PF: physical functioning; GH: general health; BP: bodily pain; RP: role physical; ODI: Oswestry disability index; VAS: visual analogue scale.

35 patients had concomitant low-grade spondylolisthesis and 6 had spondylolisthesis at more than 1 level. Spondylolisthesis was most common at the L4–L5 level, where 27 patients had a mean olisthesis of 6.2 mm (SD 2.5), followed by the L3–L4 level, where 9 patients had a mean olisthesis of 5.7 mm (SD 2.1). The third most common level was L2–L3, where 5 patients had a mean olisthesis of 5 mm (SD 1.9). At the L5–S1 level, 4 patients had olisthesis of mean 5.5 mm (SD 2.4). 1 patient had 3 mm of olisthesis at the L1–L2 level.

Spondylolisthesis was more common in women (27) than in men (8) (r_s_ = 0.35; p < 0.001). The mean age of patients with spondylolisthesis was 75 years (SD 7.1), and it was 69 years (SD 10) in patients without spondylolisthesis (r_s_ = 0.27; p = 0.004).

### Pain, disability, and function

Mean preoperative leg pain on the VAS scale was 68 (SD 24) and the corresponding score for back pain was 54 (SD 28). For EQ-5D, the mean preoperative score was 0.41 (SD 0.29). The physical subscale and general health scores of the SF-36 were generally low (Table 2). Mean preoperative ODI score was 46 (SD 15).

Preoperative walking distance was subjectively recorded by 105 patients (Table 2). There was no correlation between estimated walking distance and the minimal dural sac area, multilevel stenosis, or low-grade spondylolisthesis (Table 3).

**Table 3.** The relationship between minimal dural sac area and multilevel stenosis on the one hand and functional status, pain, and EQ-5D on the other

	Minimal area **^a^**		Multilevel spinal stenosis	
	r	p-value	r	p-value
Walking distance	–0.01	0.9	–0.02	0.8
VAS leg	–0.05	0.6	–0.24	0.03
VAS low back	–0.07	0.5	–0.09	0.4
Oswestry disability index	0.03	0.8	–0.13	0.4
SF-36, bodily pain	–0.02	0.8	0.10	0.4
SF-36, physical functioning	–0.03	0.8	–0.14	0.2
SF-36, role physical	0.09	0.4	0.12	0.3
SF-36, general health	–0.11	0.3	0.08	0.5
EQ-5D	0.13	0.3	0.03	0.8

**^a^** Correlation between minimal dural sac area and all variables, controlling for number of levels involved.

### Minimal dural sac area and multilevel stenosis

Patients with low minimal dural sac area were more likely to have multilevel disease (r_s_ = –0.43; p < 0.001). Patients with three-level stenosis had less pain on the VAS scale (54 (SD 30)), than patients with one-level (70 (SD 21)) and two-level stenosis (68 (SD 23)). The mean VAS leg pain score for patients with single-level stenosis was 72 (SD 21), as compared to 64 (SD 26) for the whole group of patients with multilevel stenosis (p = 0.1). The corresponding values for back pain were 56 (SD 25) for single-level stenosis and and 51 (SD 31) for multilevel stenosis (p = 0.4). As number of stenotic levels increased, leg pain levels deteriorated (Figure). Patients with multilevel stenosis had a more favorable level of general health than patients with single-level stenosis (p = 0.04) despite smaller dural sac area in the multilevel group (p < 0.001) (Table 4).

**Figure:**
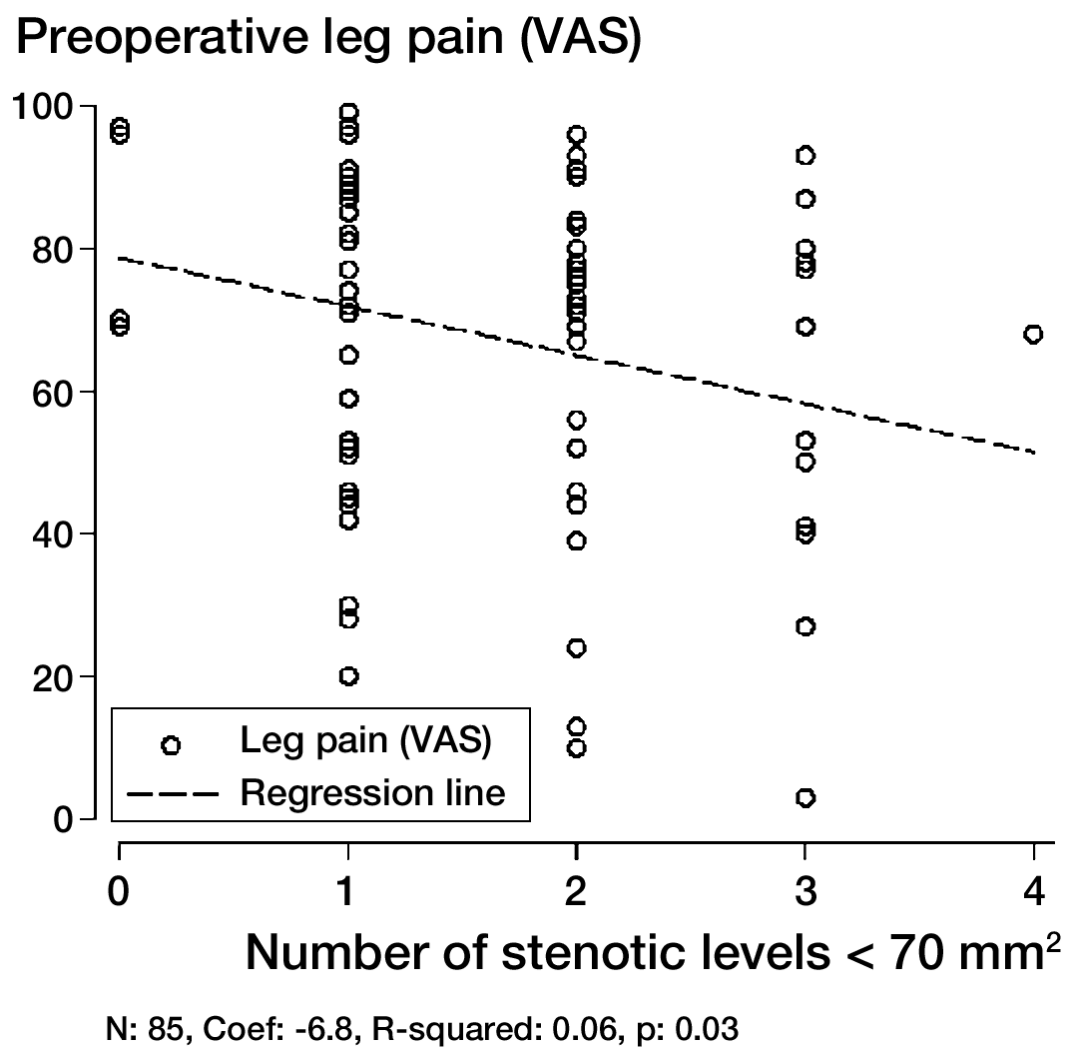
Plot of leg pain against the number of stenotic levels.

**Table 4.** Comparison of EQ-5D, functional status, and pain in patients with single and multilevel stenosis. Values are mean (SD)

	No. of patients	Age	Min. area mm^2^	EQ-5D	SF-36 PF	SF-36 RP	SF-36 BP	SF-36 GH	ODI	VAS leg	VAS back	Walking distance
Single level	55	71 (10)	48 (17)	0.45 (0.30)	35 (25)	8 (19)	26 (14)	56 (20)	48 (14)	72 (21)	56 (25)	1.7 (0.9)
Multilevel	54	71 (10)	37 (14)	0.37 (0.28)	28 (15)	11 (24)	27 (16)	64 (20)	42 (16)	64 (26)	51 (31)	1.7 (0.7)
p-value		0.82	<0.001	0.22	0.13	0.56	0.73	0.04	0.12	0.13	0.40	0.87

### Age

The minimal dural sac area became increasingly reduced with increasing age (r_s_ = –0.21; p = 0.03). The same kind of relationship was not observed between age and multilevel stenosis (r_s_ = 0.05; p = 0.7). Visual analog scores for leg or back pain were not related to age (r_s_ = 0.10; p = 0.3 and r_s_ = 0.07; p = 0.50). Older patients had shorter percieved walking distance (p = 0.07).

### Gender

30 women had multilevel disease, as compared to 24 men (p = 0.05). Low-grade spondylolisthesis was 5 times more common in women (CI: 2–13; p < 0.001). There was a difference in the degree of stenosis between the sexes, although this was not statistically significant (p = 0.07) (Table 2). Women had a lower HRLQoL in terms of EQ-5D score (0.35 as compared to 0.47 for men), but the significance of this was borderline (p = 0.07).

### Comorbidity

53 patients answered questions on comorbidity as a factor affecting quality of life. 31 reported no other disease affecting quality of life, but 22 named heart disease as a factor affecting their quality of life.

### Concomitant spondylolisthesis

The minimal dural sac area in patients with low-grade spondylolisthesis was 38 mm^2^ (SD 16), as compared to 45 mm^2^ (SD 17) in patients without spondylolisthesis (r_s_ = –0.20; p = 0.04). The mean number of levels with stenosis in patients with low-grade spondylolisthesis was 1.8 (SD 0.82) as compared to 1.5 (SD 0.76) in patients without spondylolisthesis (r_s_ = 0.18; p = 0.06).

No difference was found in visual analog score values for leg and back pain, EQ-5D, and the physical dimensions of SF-36 in patients with and without spondylolisthesis (Table 5).

**Table 5.** Age, minimal dural sac area, multilevel stenosis, and HRLQoL in patients with and without spondylolisthesis. Values are mean (SD)

Spondyl-olisthesis	No. of patients	Age	Min. area mm^2^	Multilevel stenosis	EQ-5D	SF-36 PF	SF-36 RP	SF-36 BP	SF-36 GH	ODI	VAS leg	VAS back
Yes	35	75 (7)	38 (16)	1.8 (0.82)	0.41 (0.30)	25 (14)	11 (28)	28 (13)	60 (18)	44 (14)	68 (22)	52 (27)
No	74	69 (11)	49 (25)	1.4 (0.81)	0.39 (0.29)	33 (23)	9 (19)	25 (16)	60 (21)	46 (15)	67 (25)	55 (29)
p-value		0.004	0.04	0.06	0.89	0.54	0.50	0.88	0.11	0.44	0.32	0.95

## Discussion

Our cohort of patients is unique, as we could obtain well-documented data on preoperative HRLQoL, functional status, pain, and measured MRI characteristics.

Our main findings are the high disability, low HRQoL, and high age of this population of patients, and that a very small dural sac area is more common in multilevel disease and in spondylolisthesis. We have also confirmed that there is an increased prevalence of spondylolisthesis in women (Newman 1963) and we have shown that multilevel stenosis is more prevalent in women, as are smaller dural sac area and lower HRLQoL (as measured by EQ-5D).

Our results for the physical dimensions of the SF-36 are similar to the preoperative results reported by Zanoli et al. (2006) for patients with various low back conditions (including spinal stenosis) from the same institution, which hints at stable patient selection patterns over time. The outcome measured in terms of SF-36 and EQ-5D was much lower than that of the background population in Sweden (Burström et al. 2001, Sullivan and Karlsson 1998). The low general health score (SF-36) was probably a result of a high degree of pain, and low functional ability in this group of patients and can be attributed to the lumbar spinal stenosis. SF-36 score can, however, be influenced by other factors such as comorbidity (Slover et al. 2006). The EQ-5D index scores are similar to those in a recent study from the Swedish Spine Register for outcome in surgery for spinal stenosis, where a very low score (0.36) was registred preoperatively (Jansson et al. 2009). In our study, EQ-5D score did not correlate with degree or number of stenosis. Women, however, having smaller dural sac area and a higher likelihood of spondylolisthesis, had lower EQ-5D scores than men. The lower EQ-5D score in women may well be due to more extensive degenerative changes in the female spine. Burstöm et al. (2001) showed that Swedish women between 60 and 69 have substantially lower EQ-5D scores than men of the same age group.

Ogikubo et al. (2007) have reported a positive correlation between high preoperative VAS and small cross-sectional area of the spine. In our study, patients reported high pain scores for leg and back pain. We did, however, not find a correlation between leg and back pain scores and the size of the dural sac area.

Multilevel spinal stenosis is common in the degenerative spine, which is confirmed by our study where 50% of patients had more than one level with dural sac area of < 70 mm^2^. Experimental studies have shown reduced blood flow in nerve roots in two-level, experimentally induced spinal stenosis (Takahasi et al. 1993, Jespersen et al. 1995) and Olmarker and Rydevik (1992) showed that double-level compression of the cauda equina has a more pronounced effect on nerve conduction than single-level compression. Based on clinical and imaging studies, it has been suggested that neurogenic claudication is generally associated with at least two levels of stenosis (Porter and Ward 1992). In a frequently cited article, Porter and Ward (1992) suggested that investigators should study the clinical importance of “significant two-level block” of the cauda equina with clinical parameters compared to “significant one-level block” as we have now done in this study. Hamanishi et al. (1994) reported that neurogenic claudication is associated with a cross-sectional area of < 100 m^2^ at more than 2 of 3 intervertebra levels; however, multilevel affection was rare in patients with radicular-type pain (Hamanishi et al 1994). Sato and Kikuchi (1997) found that patients with two-level stenosis more often had cauda equina symptoms than those with one-level stenosis; however, they found it uncommon for both levels to be symptomatic. In our study, leg pain decreased somewhat as the number of levels with stenosis increased. No difference between estimated walking distance in the single or multilevel groups could be found, however. Somewhat surprisingly, patients with multilevel stenosis had statistically significantly higher scores in the general health dimension of the SF-36 (better general health), which can perhaps be explained by less back and leg pain in the multilevel stenosis group. This is somewhat confusing, as the multilevel stenosis group had a smaller dural sac area. Perhaps it is the radicular-type pain in single-level stenosis and not the neurgenic claudicatio that leads to poorer general health. Our results may support the results of Sato and Kikuchi (1997) and Hamanishi et al. (1994) indicating that in multilevel stenosis, not all levels are symptomatic even with very small dural sac area and radicular-type pain is more frequent in single-level disease. Related to our results are the findings of Park et al. (2010) who, in a recent report from the SPOR trail, showed that pseudoclaudicatio is more frequent in three-level stenosis than in one- or two-level stenosis in patients without degenerative spondylolisthesis, and patients with three-level stenosis had less bodily pain. In the SPOR trail, pain radiation was less in the three-level group without degenerative spondylolisthesis (DS), and patients with two-level stenosis and DS had less pain radiation than the one-level group (Park et al. 2010). Our findings and the recent results from SPORT show that patients with multilevel disease and smaller dural sac area do indeed have less pain preoperatively than patients with single-level disease, but the explanation for this phenomen remains obscure.

Studies on the relationship between ODI and spinal stenosis have yielded varied results, and in our study ODI scores were not statistically significantly affected by spondylolisthesis, multilevel disease, or the degree of stenosis. Yukawa et al. (2002) showed ODI to be related to the degree of stenosis. However, in recent studies other authors have not found any correlation between central and lateral recess stenosis on the one hand (as evaluated by MRI) and ODI score or preoperative clinical symptoms on the other (Geisser et al. 2007, Sirvanci et al. 2008).

Subjectively estimated walking distance in patients with spinal stenosis is probably somewhat inaccurate, and can be influenced by factors other than spinal stenosis, and is therefore unspecific (Okoro et al. 2010). In the pre-MRI era, Jönsson et al. (1997) found that preoperative reduction of walking capacity tended to correlate with the width of the spinal canal, and recently Ogikubo et al. (2007) have shown correlations between pain and walking distance on the one hand and cross-sectional area of the spinal canal on the other. Patients with clinical spinal stenosis usually report reduced walking distance, as was the case in our study, and this should intuitively be related to the degree of and number of levels with morphological stenosis, although this relationship could not be found here, perhaps due to the inaccuracy of self-estimated walking distance or to the fact that the whole cohort had reached the critical stenotic level of Schönström at baseline.

We found no difference in the baseline HRLQoL indices for patients with spinal stenosis with and without spondylolisthesis, which corresponds to recently published data (Pearson et al. 2010). We found differences in the MRI characteristics of patients with spinal stenosis with and without degenerative olisthesis (DS) as patients with olisthesis were older, had smaller dural sac area, and more frequently had multilevel stenosis. Despite this, degenerative olisthesis did not make the symptoms of spinal stenosis more severe. The lack of any difference between patients with and without degenerative spondylolisthesis in preoperative HRLQoL, functional status, and pain is difficult to explain as patients with DS more often had multilevel disease and smaller dural sac area. No patients in this material had fusion, which could bias the material as it can be argued that patients with spinal stenosis and olisthesis subsequently undergoing fusion could have even more pain and even lower HRLQoL.

Why is there a lack of correlations between the hard pathomorphological data of the MRI and the outcome tools we used? The answer can perhaps be found in the critical size defined by Schönström (1988), as in this study almost all patients had reached “critical” symptomatic stenosis, profoundly affecting function and quality of life and further deterioration in dural sac area therefore unlikely to further influence quality of life indices, pain, and function. The strength of this study is that this cohort of patients comprises elderly subjects with long duration of symptoms, high pain intensity, pronounced disability, low quality of life, small dural sac area, and multilevel disease. These are all physical aspects, which would be expected to reduce the risk of surgeons' selection bias.

## References

[CIT0001] Boden SD, Davis DO, Dina TS, Patronas NJ, Wiesel SW (1990). Abnormal magnetic-resonance scans of the lumbar spine in asymptomatic subjects. J Bone Joint Surg (Am).

[CIT0002] Burström K, Johannesson M, Diderichsen F (2001). Swedish population health-related quality of life using the EQ-5D. Qual Life Res.

[CIT0003] EuroQol Group (1990). EuroQo: A new facility for the measurement of health-related quality of life. Health Policy.

[CIT0004] Fairbanks JC, Couper J, Davies JB, O'Brien JP (1980). The Oswestry low back pain disability questionnaire. Physiotherapy.

[CIT0005] Geisser ME, Haig AJ, Tong HC, Yamakawa KS, Quint DJ, Hoff JT, Miner JA, Phalke VV (2007). Spinal canal size and clinical symptoms among persons diagnosed with lumbar spinal stenosis. Clin J Pain.

[CIT0006] Hamanishi C, Matukura N, Fujita M, Tomihara M, Tanaka S (1994). Cross-sectional area of the lumbar dural tube measured from the transverse views of magnetic resonance imaging. J Spinal Disord.

[CIT0007] Jansson KA, Nemeth G, Granath F, Jönsson B, Blomqvist P (2009). Health-related quality of life (EQ-5D) before and one year after surgery for lumbar spinal stenosis. J Bone Joint Surg (Br).

[CIT0008] Jespersen SM, Hansen ES, Hoy K, Christensen KO, Lindblad BE, Ahrensberg J, Bunger C (1995). Two-level spinal stenosis in minipigs: hemodynamic effects of exercise. Spine.

[CIT0009] Jönsson B, Annertz M, Sjoberg C, Strömqvist B (1997). A prospective and consecutive study of surgically treated lumbar spinal stenosis. Part I: Clinical features related to radiographic findings. Spine.

[CIT0010] Malmivaara A, Slatis P, Heliovaara M (2007). Surgical or nonoperative treatment for lumbar spinal stenosis? A randomized controlled trail. Spine.

[CIT0011] Newman PH (1963). The etiology of spondylolisthesis. J Bone Joint Surg (Br).

[CIT0012] Ogikubo O, Forsberg L, Hansson T (2007). The relationship between the cross-sectional area of the cauda equina and the preoperative symptoms in central lumbar spinal stenosis. Spine.

[CIT0013] Okoro T, Qureshi A, Sell B, Sell P (2010). The accuracy of assessment of walking distance in the elective spinal outpatients setting. Eur Spine J.

[CIT0014] Olmarker K, Rydevik B (1992). Single- versus double-level nerve root compression. An experimental study on the porcine cauda equina with analyses of nerve impulse conduction properties. Clin Orthop.

[CIT0015] Park DK, An HS, Lurie JD, Zaho W, Tosteson A, Tosteson TD, Herkowitz H, Errico T, Weinstein JN (2010). Does multilevel lumbar stenosis lead to poorer outcomes? A subanalysis of the spine patient outcomes research trial (SPORT) lumbar stenosis study. Spine.

[CIT0016] Pearson A, Blood E, Lurie J, Tosteson T Abdu WA, Hillibrand A, Bridwell K, Weinstein JN (2010). Degenerative Spondylolisthesis Versus Spinal Stenosis: Does a Slip Matter? Comparison of Baseline Characteristics and Outcomes (SPORT). Spine.

[CIT0017] Porter RW, Ward D (1992). Cauda equina dysfunction. The significance of two-level pathology. Spine.

[CIT0018] Sato K, Kikuchi S (1997). Clinical analysis of two-level compression of the cauda equina and the nerve roots in spinal canal stenosis. Spine.

[CIT0019] Schönström NSR (1988). The narrow lumbar canal and the size of the cauda equina in man.

[CIT0020] Sirvanci M, Bhatia M, Ganiyusufoglu KA, Duran C, Tezer M, Ozturk C, Aydogan M, Hamzaoglu A (2008). Degenerative lumbar spinal stenosis: correlation with Oswestry Disability Index and MR imaging. Eur Spine J.

[CIT0021] Slover J, Abdu WA, Hanscom B, Weinstein JN (2006). The impact of comorbidities on the change in Short-Form 36 and Oswestry scores following lumbar spine surgery. Spine.

[CIT0022] Strömqvist B, Fritzell P, Hägg O, Jönsson B (2009). The Swedish Spine Register: development, design and utility. Eur Spine J.

[CIT0023] Sullivan M, Karlsson J (1998). The Swedish SF-36 Health Survey III. Evaluation of criterion-based validity: results from normative population. J Clin Epidemiol.

[CIT0024] Takahashi K, Olmarker K, Holm S, Porter RW, Rydevik B (1993). Double-level cauda equina compression: an experimental study with continuous monitoring of intraneural blood flow in the porcine cauda equina. J Orthop Res.

[CIT0025] Ware JE jr, Sherbourne CD (1992). The MOS 36-item short-form health survey SF-36 I. Conceptual framework and item selection. Med Care.

[CIT0026] Yukawa Y, Lenke LG, Tenhula J, Bridwell KH, Riew D, Blanke K (2002). A comprehensive study of patients with surgically treated lumbar spinal stenosis with neurogenic claudication. J Bone Joint Surg (Am).

[CIT0027] Zanoli G, Jönsson B, Strömqvist B (2006). SF-36 scores in degenerative lumbar spine disorders. Analysis of prospective data from 451 patients. Acta Orthop.

